# High temporal resolution RNA-seq time course data reveals widespread synchronous activation between mammalian lncRNAs and neighboring protein-coding genes

**DOI:** 10.1101/gr.276818.122

**Published:** 2022-08

**Authors:** Walter Muskovic, Eve Slavich, Ben Maslen, Dominik C. Kaczorowski, Joseph Cursons, Edmund Crampin, Maria Kavallaris

**Affiliations:** 1 Children's Cancer Institute, Lowy Cancer Research Centre, University of New South Wales, Sydney, New South Wales 2052, Australia;; 2 ARC Centre of Excellence in Convergent Bio-Nano Science and Technology, Australian Centre for NanoMedicine, University of New South Wales Australia, Sydney, New South Wales 2052, Australia;; 3 School of Clinical Medicine, University of New South Wales Medicine and Health, University of New South Wales Sydney, Sydney, New South Wales 2052, Australia;; 4 Stats Central, Mark Wainwright Analytical Centre, University of New South Wales, Sydney, New South Wales 2052, Australia;; 5 Garvan Institute of Medical Research, Sydney, New South Wales 2010, Australia;; 6 The Department of Biochemistry and Molecular Biology, Biomedicine Discovery Institute, Monash University, Clayton, Victoria 3800, Australia;; 7 Bioinformatics Division, The Walter and Eliza Hall Institute of Medical Research and Department of Medical Biology and Faculty of Medicine, Dentistry and Health Sciences, University of Melbourne, Parkville, Victoria 3052, Australia;; 8 Systems Biology Laboratory, School of Mathematics and Statistics and Department of Biomedical Engineering, University of Melbourne, Victoria 3010, Australia;; 9 ARC Centre of Excellence in Convergent Bio-Nano Science and Technology, Melbourne School of Engineering, University of Melbourne, Parkville, Victoria 3010, Australia

## Abstract

The advent of massively parallel sequencing revealed extensive transcription beyond protein-coding genes, identifying tens of thousands of long noncoding RNAs (lncRNAs). Selected functional examples raised the possibility that lncRNAs, as a class, may maintain broad regulatory roles. Expression of lncRNAs is strongly linked with adjacent protein-coding gene expression, suggesting potential *cis*-regulatory functions. A more detailed understanding of these regulatory roles may be obtained through careful examination of the precise timing of lncRNA expression relative to adjacent protein-coding genes. Despite the diversity of reported lncRNA regulatory mechanisms, where causal *cis*-regulatory relationships exist, lncRNA transcription is expected to precede changes in target gene expression. Using a high temporal resolution RNA-seq time course, we profiled the expression dynamics of several thousand lncRNAs and protein-coding genes in synchronized, transitioning human cells. Our findings reveal that lncRNAs are expressed synchronously with adjacent protein-coding genes. Analysis of lipopolysaccharide-activated mouse dendritic cells revealed the same temporal relationship observed in transitioning human cells. Our findings suggest broad-scale *cis*-regulatory roles for lncRNAs are not common. The strong association between lncRNAs and adjacent genes may instead indicate an origin as transcriptional by-products from active protein-coding gene promoters and enhancers.

Large-scale transcriptomic studies, enabled by improvements in total RNA enrichment and high-throughput RNA profiling technologies, unexpectedly revealed extensive transcription outside the boundaries of known protein-coding genes (Kapranov et al. 2002; Okazaki et al. 2002; The FANTOM Consortium and RIKEN Genome Exploration Research Group and Genome Science Group 2005; Djebali et al. 2012; The ENCODE Project Consortium et al. 2020). The class of products of this transcription are now known as long noncoding RNAs (lncRNAs). Throughout the human genome, tens of thousands of these transcripts have been accurately annotated (Derrien et al. 2012; Hon et al. 2017). Despite their ubiquity, the biological significance of most lncRNAs remains unknown.

However, three consistently documented properties of these transcripts hint at potential widespread regulatory roles. Firstly, whereas lncRNA exon sequences are poorly conserved, their promoter region sequences are conserved at levels equivalent to protein-coding genes (The FANTOM Consortium and RIKEN Genome Exploration Research Group and Genome Science 2005; Guttman et al. 2009; Derrien et al. 2012; Chen et al. 2016). Second, lncRNAs display exquisite tissue specificity in their expression patterns (Cabili et al. 2011; Derrien et al. 2012; Djebali et al. 2012). Thirdly, lncRNA expression is often closely correlated with neighboring protein-coding genes, both in developing (Ponjavic et al. 2009; Herriges et al. 2014; Sarropoulos et al. 2019) and adult tissues (Derrien et al. 2012; Luo et al. 2016; Hon et al. 2017). Taken together, these observations suggest that lncRNA transcription may serve a functional role in promoting activation of tissue-specific, adjacent protein-coding genes.

Reported lncRNA *cis*-regulatory mechanisms are diverse. Detailed investigation of individual lncRNAs has revealed their involvement in the recruitment of regulatory factors and chromatin remodeling complexes through direct RNA–protein interaction, whereas in other cases the process of transcription itself appears to be sufficient, by either increasing the local concentration of transcription-associated factors or establishing a permissive chromatin state (Wang et al. 2011; Cabianca et al. 2012; Anderson et al. 2016; Engreitz et al. 2016). Although these studies of individual lncRNAs are illuminating, a genome-wide approach is required to establish the generality of each mechanism. To address this need, we turned to a relatively understudied dimension of regulatory RNA activity; the kinetics associated with their proposed *cis*-regulatory mechanisms. Given their nature as noncoding transcripts, a causal relationship suggests that lncRNA transcription should precede changes in target gene expression—whether through recruitment of regulatory factors or facilitating a more permissive local chromatin structure. As transcription kinetics are slow (Tennyson et al. 1995; Fuchs et al. 2014; Jonkers et al. 2014), relative to the rapid activation of inducible transcription factors (Hager et al. 2009; Fowler et al. 2011), gene expression measurements of sufficient granularity should reveal the timing of lncRNA transcription relative to target gene activation. Such detailed dynamic information may provide insight into the most likely mechanisms of action. Indeed, existing limited investigations of lncRNA dynamics in transitioning mammalian cells indicate that lncRNA production precedes activation of protein-coding genes (De Santa et al. 2010; Aitken et al. 2015; Arner et al. 2015), providing evidence for lncRNAs as ubiquitous *cis*-regulators of gene expression. However, these investigations have relied on cap analysis of gene expression (CAGE), which captures only the 5′ end of a transcript, or have used time series of limited duration and resolution.

Here, we aim to use high temporal resolution rRNA-depleted total RNA-seq measurements to capture the genome-wide dynamics of lncRNAs and protein-coding genes in transitioning mammalian cells. Using these data, we will investigate the temporal hierarchy of lncRNA and protein-coding gene activation to assess the feasibility of broad-scale *cis*-regulatory roles for lncRNAs.

## Results

### Capturing a dynamic transcriptome at high temporal resolution

**Figure 1.**
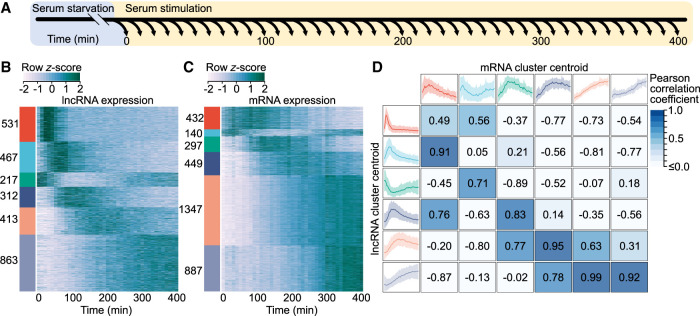
Protein-coding genes and lncRNAs exhibit distinct expression dynamics. (*A*) Schematic representation of the experimental design. Following stimulation, cells were harvested at evenly spaced 10-min intervals, yielding a total of 41 time points. (*B*) Heat map of lncRNA expression. Each row represents an individual *z*-score-normalized lncRNA expression profile. Colored bars indicate six clusters obtained through *k*-means cluster analysis, labeled with the number of transcripts in each. (*C*) Heat map of mRNA expression, as in *B*. (*D*) Comparison of lncRNA and mRNA cluster centroids. *Outer* boxes display cluster centroids, capturing the mean expression of all cluster members. Shaded regions represent the 5th–95th percentiles of all cluster member expression profiles. Pearson correlation coefficients, displayed in the *center* boxes, were calculated between all lncRNA and mRNA centroid expression profiles.

To capture lncRNA and protein-coding gene transcription dynamics at high temporal resolution, a reliable method to obtain a homogeneous, synchronized cell population was required. To achieve this, we took advantage of the unique growth characteristics of the immortalized human glioblastoma cell line T98G. T98G cells retain growth arrest mechanisms characteristic of untransformed cells (Stein 1979). In response to growth factor deprivation, T98G cells undergo reversible G_0_/G_1_ cell cycle arrest. Serum stimulation is sufficient to induce exit from growth arrest, producing a population of tightly synchronized cycling cells, without the need for drug treatment (Canhoto et al. 2000; Takahashi et al. 2000; Tullai et al. 2007). Following stimulation, the transition from quiescence to active cell division is characterized by the induction of a complex transcriptional cascade involving protein synthesis-independent induction of immediate early genes, followed by synthesis-dependent secondary response genes (Tullai et al. 2007). To capture this transcriptional program at high temporal resolution, synchronized transitioning T98G cells were sampled at 10-min intervals, from 0 min (unstimulated) to 400 min (Fig. 1A).

To obtain gene expression estimates, rRNA-depleted total RNA-seq was performed for all time points. Examination of genome-aligned sequencing reads revealed a large number of lncRNAs were missing from existing genome annotations. To overcome this, de novo transcriptome assembly was performed (see Methods), identifying 2803 lncRNAs in addition to 3552 protein-coding genes activated in response to serum stimulation. Of the identified lncRNAs, 33.2% had no overlap with either GENCODE (Derrien et al. 2012) or FANTOM CAT (Hon et al. 2017) annotated lncRNA transcripts. Notably, 998 lncRNAs exhibited a rapid increase in expression, peaking within the first 100 min of stimulation, followed by an equally rapid decrease in expression (Fig. 1B). In contrast, protein-coding mRNAs displayed more gradual dynamics, with most mRNAs accumulating progressively throughout the time course (Fig. 1C). To directly compare lncRNA and mRNA expression dynamics, we examined the correlation between the prototypical responses displayed by the two transcript classes (Fig. 1D). Notably, coding genes lacked the early rapid response exhibited by 998 lncRNAs, consistent with previous observations of lncRNAs preceding the expression of protein-coding genes in transitioning mammalian cells (De Santa et al. 2010; Aitken et al. 2015; Arner et al. 2015).

However, we noted that activated protein-coding genes were significantly longer than the class of lncRNAs (Supplemental Fig. 1). Longer transcription times could introduce delays in mature mRNA accumulation. Protein-coding mRNA half-lives are also known to vary over a wide range, whereas lncRNAs are generally rapidly degraded by the RNA exosome (Preker et al. 2008; Schlackow et al. 2017). The combination of gene length and mRNA stability may mask the time of transcription initiation of protein-coding genes (gene activation), impeding accurate comparison with lncRNA activation dynamics. To determine if these effects were obscuring the true protein-coding gene induction times, we next examined the contributions of these two factors to mRNA expression dynamics.

### Transcript stability shapes mRNA expression dynamics

**Figure 2.**
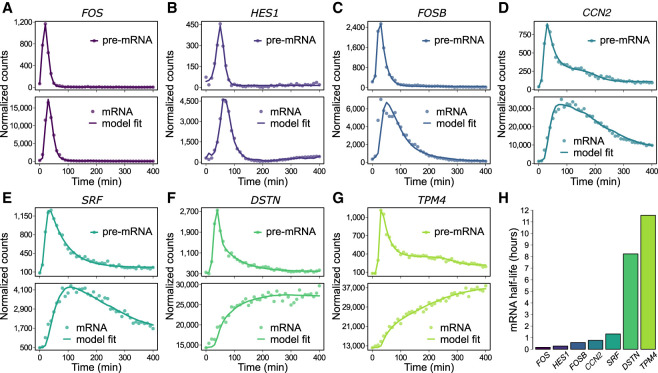
Gene-specific degradation rates shape mRNA dynamics. (*A*–*G*) Pre-mRNA (*top* panels) and mRNA expression profiles (*bottom* panels) of seven representative genes with rapid pre-mRNA dynamics. Pre-mRNA and mRNA expression profiles (points) were obtained by quantification of RNA-seq reads mapping to gene introns and exons, respectively. Pre-mRNA expression profiles are overlaid with impulse model fits (lines) to aid visualization. mRNA expression profiles are overlaid with the transcription model fits (lines) used to obtain gene-specific mRNA half-lives, presented in *H*.

To gain a quantitative understanding of the effect of transcript stability on measured mRNA dynamics, we adapted a mathematical model of the transcriptional response proposed by Zeisel et al. (2011) (see Methods), in which the rate of change of mRNA concentration is determined by a balance between mRNA degradation and the production of new mRNA from unspliced precursor-mRNA (pre-mRNA). RNA-seq reads originating from intronic regions and captured in total RNA-seq have been demonstrated to serve as a useful proxy for nascent transcription (Gaidatzis et al. 2015; La Manno et al. 2018) and were used to estimate pre-mRNA concentration. Time-invariant splicing and degradation rates were selected that minimized the deviation between model predictions of mRNA concentration relative to measured levels. This model provided a close fit to observed expression dynamics (Fig. 2A–G), enabling estimation of transcript-specific half-lives (Fig. 2H).

Genes with relatively unstable mRNA largely recapitulated pre-mRNA dynamics with a short time lag. In contrast, longer mRNA half-lives resulted in expression dynamics increasingly divergent from the transient precursor. These results suggest that, for genes encoding stable transcripts, mRNA expression profiles serve as a poor indicator of underlying gene induction dynamics. Furthermore, the confounding effect of transcript stability can be avoided by measuring pre-mRNA expression dynamics for each mRNA transcript through quantification of intron-mapping RNA fragments.

### Gene length introduces RNA production delays

**Figure 3.**
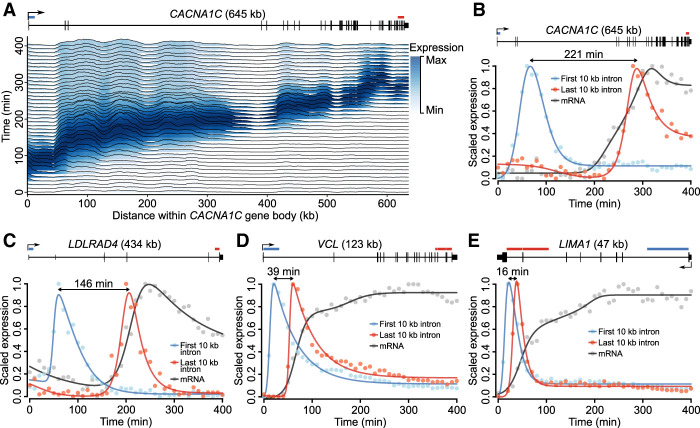
Gene length delays mRNA production. (*A*) Transcription across the *CACNA1C* gene body. Ridges display normalized RNA-seq coverage over 1-kb intervals tiled across *CACNA1C* introns. Color intensity indicates the scaled expression of each 1-kb interval across the time course. A right-facing arrow at the 5′ end of the gene schematic (*top*) indicates the direction of transcription. (*B*–*E*) mRNA and pre-mRNA expression dynamics for four genes of varying length. Pre-mRNA expression is shown for the first and last 10 kb of each gene's introns, indicated *above* each gene schematic by blue and red horizontal bars, respectively. The approximate delay between transcription of the first and last 10 kb of pre-mRNA is indicated by a left-right arrow between the two expression profile peaks. Expression profiles are overlaid with impulse model fits (lines) and scaled to values between zero and one to facilitate visual comparison.

Human gene length varies over a wide range (Supplemental Fig. 1). Protein-coding genes identified in this study ranged from less than 1 kb to more than 1 Mb in length, with a mean length of 51.8 kb. In contrast, lncRNAs were observed to be significantly shorter than most protein-coding genes, consistent with previous annotations (Cabili et al. 2011; Derrien et al. 2012; Hon et al. 2017), with a mean length of 16.6 kb (Supplemental Fig. 1). The time required for Pol II to complete transcript elongation may delay the production of mature mRNA. These effects are expected to be more pronounced for longer genes. This was seen to be the case for the *CACNA1C* gene (Fig. 3A). Visualization of RNA-seq coverage over intronic regions revealed a progressive wave of transcription across the length of the 645 kb gene. Mature mRNA production is correspondingly observed to be delayed by several hours (Fig. 3B). Examination of shorter genes revealed delays in mature mRNA accumulation due to transcriptional delays in proportion to gene length (Fig. 3C–E).

From these data, we estimated transcription elongation to proceed at a rate of ∼2.5 kb/min (Supplemental Fig. 2), in line with previous estimates (Tennyson et al. 1995; Fuchs et al. 2014; Jonkers et al. 2014). Assuming this constant rate, the time required to complete transcription elongation of an average length protein-coding gene is ∼21 min. These results suggest that mature mRNA expression profiles may be a poor indicator of induction dynamics, particularly for long genes. Further, to negate the effects of transcription delays due to gene length, RNA-seq reads originating from the 5′ end of a gene's pre-mRNA would be most suitable for determining the timing of gene activation.

### mRNA expression masks underlying gene induction dynamics

**Figure 4.**
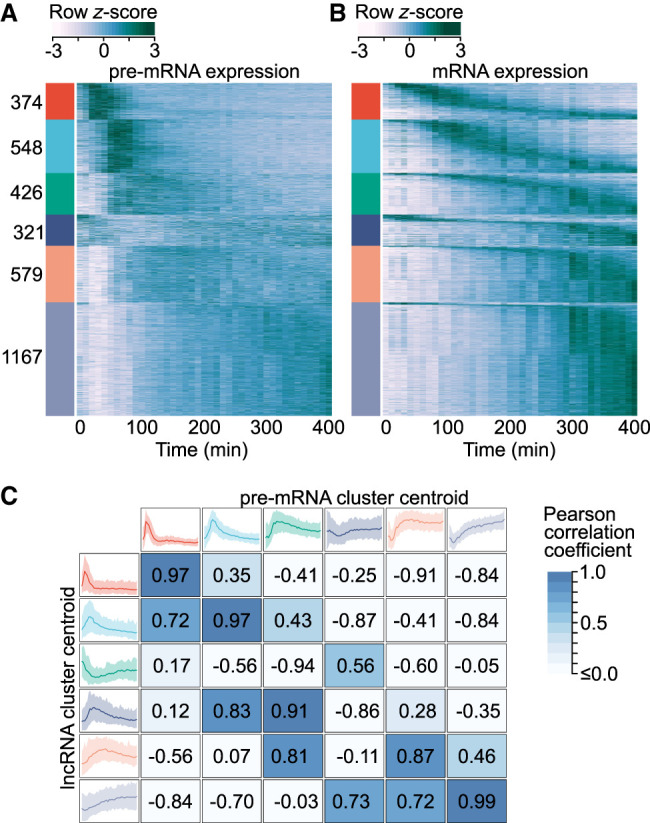
mRNA expression fails to capture gene induction dynamics. (*A*) Heat map of protein-coding gene induction dynamics. Expression profiles were captured using the first 10 kb of gene introns and *z*-score normalized. Colored bars (*left*) indicate cluster membership to one of six clusters obtained through *k*-means cluster analysis. Clusters are labeled with the number of transcripts in each. (*B*) Heat map of protein-coding mRNA expression dynamics. Rows within each expression cluster are ranked by the time of peak expression. Rows within *A* and *B* correspond to the same genes. (*C*) Comparison of protein-coding gene pre-mRNA and lncRNA expression dynamics. lncRNA cluster centroids (*left*) are the same as in Figure 1B, whereas protein-coding pre-mRNA centroids (*top*) correspond to the colored bars in *B*. Centroids represent the mean expression of all cluster members, whereas shaded regions represent the 5th–95th percentiles. Pearson correlation coefficients calculated between all lncRNA and protein-coding pre-mRNA centroids are presented.

Taken together, our findings suggest that the combined effects of gene length and transcript-specific degradation rates may combine to mask protein-coding gene induction dynamics. To remove the contributions of these effects, gene expression profiles were quantified for all protein-coding and lncRNA transcripts using only the expression of the first 10 kb of intron sequence. These gene expression measurements were used for all future analyses. Pre-mRNA profiles (Fig. 4A) revealed that protein-coding gene activation is significantly more rapid than indicated by mature mRNA expression levels (Fig. 1C). Within each pre-mRNA expression cluster, genes were ordered by their mRNA expression dynamics (Fig. 4B). Genes with similar pre-mRNA profiles produced a broad range of mature mRNA dynamics, suggesting that the combined effects of gene length and transcript stability shape protein-coding gene expression dynamics.

We next compared the prototypical responses revealed by pre-mRNA with the expression profiles characteristic of lncRNAs (Fig. 4C). In contrast to the relationship implied by mature mRNA expression (Fig. 1D), pre-mRNA dynamics revealed that the rapid responses exhibited by lncRNAs are also observed for the induction of protein-coding genes.

### LncRNAs mirror adjacent protein-coding gene expression

**Figure 5.**
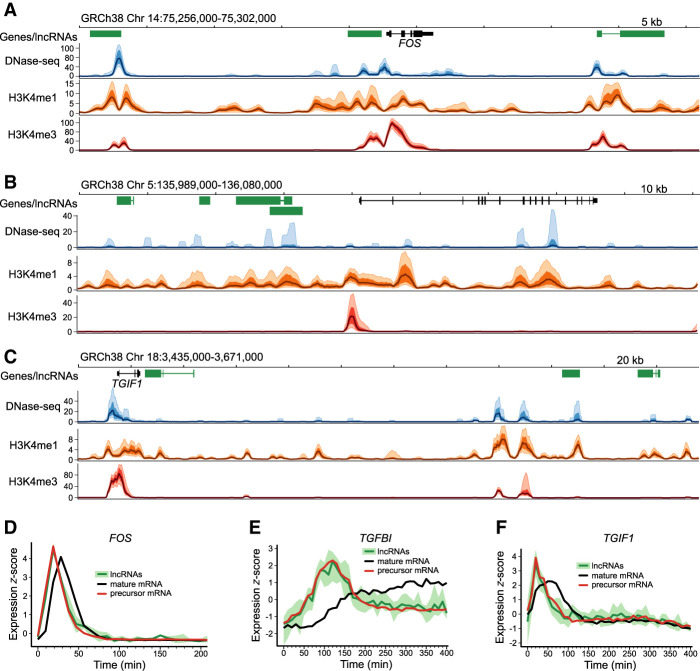
Human lncRNAs mirror adjacent protein-coding gene pre-mRNA expression. (*A*–*C*) NIH Roadmap Epigenomics data for loci surrounding protein-coding genes; FOS, TGFBI, and TGIF1. A schematic of each locus is presented, with GENCODE-annotated protein-coding genes shown in black and lncRNAs in green. NIH Roadmap Epigenomics DNase-seq, H3K4me1, and H3K4me3 histone modification ChIP-seq data from 111 uniformly processed human epigenomes are presented. Lines depict mean −log_10_(*P*-value) signal, with dark shaded regions indicating 25%–75% percentiles, and lighter shaded regions the 10%–90% percentiles. (*D*–*F*) Line plots of *z*-score normalized protein-coding gene and lncRNA expression values. LncRNA and pre-mRNA were quantified using the expression of the first 10 kb of intronic regions. Mean expression (dark green) and the range of all expression values (shaded light green) is shown for adjacent lncRNAs. Mature mRNA expression is included for comparison.

Having identified that lncRNAs and protein-coding genes exhibit similar dynamics, we next sought to examine the spatial relationship between lncRNAs and the expression profiles of adjacent protein-coding genes. Before examining the genome-wide relationship, we focused in detail on three well-studied genes activated early in the release from cell cycle arrest (Fig. 5).

We first considered the proto-oncogene *FOS*. Following serum stimulation, canonical mitogen-activated protein kinase signaling triggers rapid transcription of immediate early genes, including *FOS* (Angel and Karin 1991). The encoded transcription factor subunit, FOS, dimerizes with JUN to form the transcriptional activator AP-1, stimulating further downstream transcriptional changes. Examination of RNA-seq data from the *FOS* locus revealed rapid and transient transcription of *FOS* and two adjacent lncRNAs. Both lncRNAs were associated with regions of increased nuclease sensitivity, revealed by a strong DNase-seq signal across diverse human tissues (Fig. 5A). These regions also overlapped H3K4me1 and H3K4me3 histone marks characteristic of enhancer regions (Ernst et al. 2011; Roadmap Epigenomics Consortium et al. 2015). The expression profiles of both lncRNAs were captured and compared with the adjacent protein-coding *FOS*. Despite the rapid dynamics exhibited within this group, the high temporal resolution of the RNA-seq time series allowed *FOS* pre-mRNA and mRNA dynamics to be separated. Both lncRNAs were found to mirror the expression dynamics of *FOS* pre-mRNA (Fig. 5D).

We next considered *TGFBI*, which encodes an excreted extracellular matrix protein involved in cell adhesion and migration (Fig. 5B). In contrast to the transient dynamics of *FOS*, *TGFBI* exhibited gradual accumulation and increased separation of pre-mRNA and mature mRNA expression profiles (Fig. 5E). Four lncRNAs were identified, clustered upstream of *TGFBI*. Transcription was observed to overlap enhancer-associated chromatin marks. As was observed for *FOS*, comparison of expression dynamics revealed that lncRNA expression mirrored the activation of the adjacent protein-coding gene (Fig. 5E).

As a third example, we examined the dynamics of the well-studied transcription factor gene *TGIF1*, which mediates a critical role in attenuating transforming growth factor beta pathway signaling (Wotton et al. 1999). In addition to the lncRNA antisense to *TGIF1*, two lncRNAs were identified more than 100 kb downstream (Fig. 5C). All lncRNAs overlapped chromatin marks, of variable signal intensity, characteristic of enhancer regions. Consistent with *FOS* and *TGFBI*, analysis of the expression dynamics revealed that all lncRNAs mirrored the activation of *TGIF1* (Fig. 5F).

### Protein-coding gene and lncRNA expression correlation is genome-wide and exhibits synchrony

**Figure 6.**
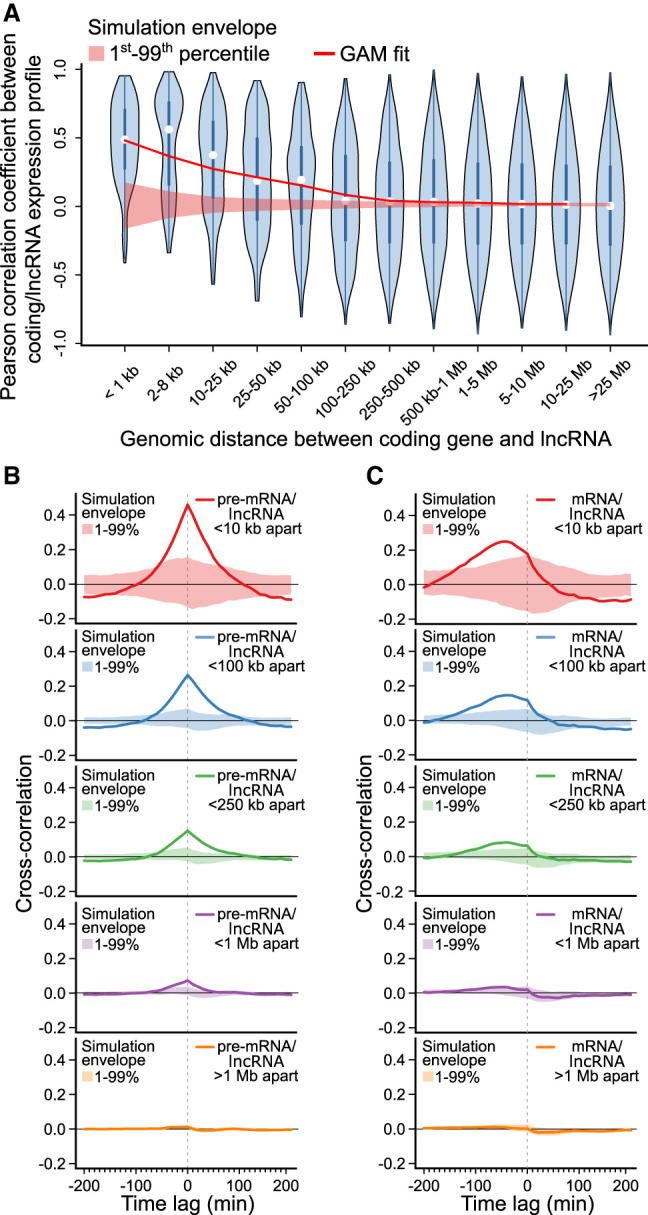
Human lncRNAs mirror adjacent protein-coding gene expression. (*A*) Violin plot of Pearson correlation coefficients between protein-coding gene and lncRNA expression profiles, binned by distance between transcripts’ transcriptional start sites. A generalized additive model (GAM) fit summarizes the relationship between distance and correlation of protein-coding/lncRNA pairs (e.d.f. = 8.197, *P* < 2^−16^). A simulation envelope, generated using a block-bootstrap approach and presented as a red shaded band (see Methods), demonstrates the expected trend under the null hypothesis that distance and correlation are unrelated. The trend in correlation against separation distance lies well outside the simulation envelope, indicating a relationship unlikely to be due to chance. Continuous GAM fit and simulation envelope values were overlaid by plotting the mean of each distance bin. (*B*) Similarity between expression profiles of coding/lncRNA distance-binned pairs, at time lags of −200–200 min. Solid lines represent the mean correlation coefficient calculated between distance-binned pairs at varying time-lags of lncRNA expression profiles relative to coding gene expression. Simulation envelopes generated using a block bootstrap approach show the expected cross-correlations versus time trends where there is no relationship with separation distance. (*C*) Produced as in *B*, with coding gene expression profiles replaced with mature mRNA expression, rather than pre-mRNA.

Close examination of *FOS*, *TGFBI*, and *TGIF1* identified adjacent lncRNAs that mirror protein-coding gene activation. To assess the generality of this phenomenon in our data, we next examined the relationship between distance and similarity in expression between all 3552 protein-coding genes and 2803 lncRNAs activated across the human genome. Consistent with observations of individual genes, lncRNAs and protein-coding genes exhibited increasing correlation with decreasing genomic distance (Fig. 6A). Consistent with previous observations (Ebisuya et al. 2008), a similar trend is observed within the two transcript classes (Supplemental Fig. 3). Accordingly, a block bootstrap approach was employed (see Methods) to assess uncertainty around the trend between distance and correlation observed between the two transcript classes. Strong deviation of the trend, summarized by the generalized additive model (GAM) fit, from the obtained confidence intervals suggests that associations between the expression of lncRNAs and adjacent protein-coding genes is generalizable across our data.

To determine whether this trend was consistent between lncRNAs uniquely identified in this study (930) and lncRNAs overlapping existing annotations (1873), the analysis was repeated separately for each group of lncRNAs. The trend between lncRNAs and adjacent protein-coding genes was observed in both groups (Supplemental Fig. 4). lncRNAs have also been classified as promoter-associated or enhancer-associated according to the chromatin status at the transcriptional initiation region (Marques et al. 2013), with emphasis that enhancer-associated lncRNAs are likely to predominately function in *cis* (Li et al. 2016; Sartorelli and Lauberth 2020). To determine whether the observed trend was consistent between promoter-associated and enhancer-associated lncRNAs, transcripts were classified based on the relative levels of histone H3K4 trimethylation and H3K27 acetylation near their transcription start sites (see Methods) and the analysis was repeated separately for each group of lncRNAs. The trend between lncRNAs and adjacent protein-coding genes was observed in both classes (Supplemental Fig. 5).

Having identified a genome-wide association between protein-coding gene and adjacent lncRNA expression, we next sought to examine the sequence of events. To determine whether lncRNA expression precedes or trails the activation of adjacent genes, time-lagged lncRNA expression profiles were compared with protein-coding pre-mRNA expression (Fig. 6B). Cross-correlation between lncRNA and protein-coding expression profiles was found to be maximal with a lag of 0 min. These results suggest that lncRNA expression and coding gene activation are synchronous, consistent with the observations of individual lncRNA–gene pairs (Fig. 5D–F). In contrast, when lncRNA and coding gene dynamics were compared using mature mRNA expression, lncRNA expression appeared to significantly precede protein-coding gene activation (Fig. 6C). These findings highlight the utility of measuring 5′ intron expression to capture gene activation dynamics and provide a possible explanation for the previously reported finding that transcription of lncRNAs precedes protein-coding gene expression (De Santa et al. 2010; Aitken et al. 2015; Arner et al. 2015).

### Murine lncRNAs mirror adjacent protein-coding gene expression

In the T98G time series data, simultaneous initiation of lncRNA and adjacent protein-coding expression is consistent across the human genome. To evaluate whether this is also the case in the mouse genome, we examined an RNA-seq time series capturing the immune response of mouse dendritic cells to lipopolysaccharide (LPS) captured at 15-min time intervals, from 0 to 180 min (Rabani et al. 2014). To identify mouse lncRNAs, de novo transcriptome assembly was again performed (see Methods), identifying 1275 lncRNAs and 2882 protein-coding genes activated in response to LPS stimulation. Of the identified lncRNAs, 34.4% had no overlap with GENCODE-annotated lncRNA transcripts.

**Figure 7.**
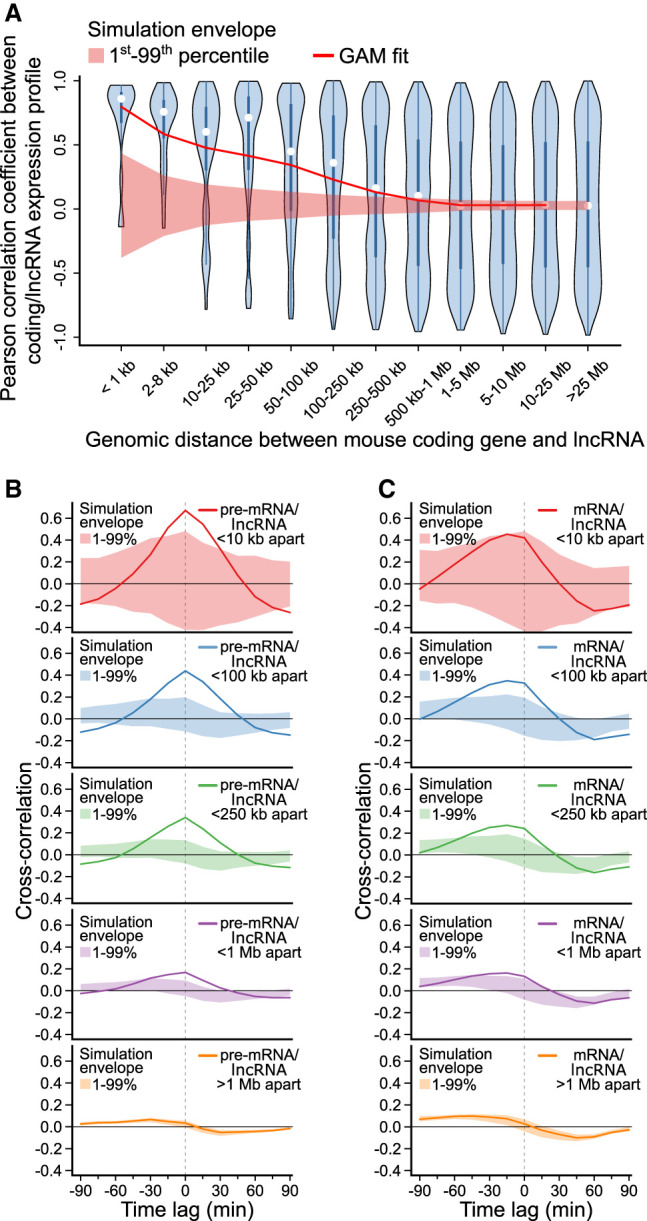
Murine lncRNAs mirror adjacent protein-coding gene expression. Spatial and temporal relationship between protein-coding genes and lncRNAs activated in mouse dendritic cells responding to stimulation with lipopolysaccharide (Rabani et al. 2014). (*A*) Violin plot of Pearson correlation coefficients between protein-coding gene and lncRNA expression profiles, binned by distance between transcripts’ transcriptional start sites. A GAM fit summarizes the relationship between distance and correlation of mouse protein-coding/lncRNA pairs (e.d.f. = 7.007, *P* < 2^−16^). A simulation envelope, presented as a red shaded band, generated using a block-bootstrap approach (see Methods) demonstrates the expected trend under the null hypothesis that distance and correlation are unrelated. The trend in correlation against separation distance lies well outside the simulation envelope indicating a relationship unlikely to be due to chance. Continuous GAM fit and simulation envelope values were overlaid by plotting the mean of each distance bin. (*B*) Similarity between expression profiles of coding/lncRNA distance-binned pairs, at time lags of from −90 to 90 min. Solid lines represent the mean correlation coefficient calculated between distance-binned pairs at varying time-lags of lncRNA expression profiles relative to coding gene expression. Simulation envelopes generated using a block bootstrap approach show the expected cross-correlations versus time trends where there is no relationship with separation distance. (*C*) Produced as in *B*, with coding gene expression profiles replaced with mature mRNA expression, rather than pre-mRNA.

Consistent with lncRNAs examined in the human T98G time series data set, mouse lncRNA expression was significantly associated with activation of adjacent protein-coding genes (Fig. 7A). Comparing lagged lncRNA gene expression with nearby protein-coding expression profiles, measured using 5′ intron expression, correlation was again found to be maximal with a time lag of 0 min (Fig. 7B). When gene expression dynamics were measured using mature mRNA, lncRNA expression appeared to precede protein-coding gene activation (Fig. 7C). These results suggest that synchronous activation of lncRNAs and neighboring protein-coding genes is a general phenomenon in transitioning mammalian cells.

## Discussion

Our findings establish a robust relationship between lncRNAs and the expression of adjacent protein-coding genes. Through genome-wide comparison of lncRNA and coding-gene activation dynamics, we have demonstrated that, within the temporal resolution of our measurements, lncRNA and protein-coding gene activation appears to be synchronous.

This observation contrasts with previous reports identifying lncRNA expression as preceding activation of protein-coding genes in transitioning mammalian cells (De Santa et al. 2010; Aitken et al. 2015; Arner et al. 2015). Our findings suggest that this discrepancy may be attributed to the reliance of previous investigations on measurement of mature mRNA to capture gene expression. We have shown that gene length introduces considerable delays in mRNA accumulation. When combined with differences in transcript stability, our results indicate that mRNA levels are an unreliable indicator of gene activation times. In contrast, we have demonstrated that measurement of pre-mRNA expression levels from RNA-seq data reliably captures the timing of gene activation.

Reports of delays between lncRNA and mRNA transcription have been interpreted as evidence supporting functional roles for lncRNAs as pervasive transcriptional regulators (De Santa et al. 2010; Schaukowitch et al. 2014; Arner et al. 2015). This reasoning is consistent with noncoding transcripts that must be transcribed prior to any regulatory activity. Regardless of the specific *cis*-regulatory mechanism employed, where a functional regulatory relationship exists in which a lncRNA activates the expression of a neighboring gene, lncRNA expression is expected to occur in advance of changes in target gene expression. Our findings indicate that, with an average length of 16.6 kb and transcription elongation rate of 2.5 kb/min, a typical lncRNA would take 6.6 min to be transcribed, excluding the time required for recruitment of regulatory complexes or other proposed *cis*-regulatory roles. The high temporal resolution of the time courses described in this study did not reveal such a delay. Instead, lncRNA and protein-coding gene activation appear to be synchronous.

These findings do not support the existence of broad-scale *cis*-regulatory roles for lncRNAs. Both human and mouse lncRNAs identified in this study arise as transient, low-abundance transcription mirroring adjacent gene activation. These observations are consistent with proposals that the majority of lncRNAs may represent the nonspecific initiation of transcription at active regulatory elements (Wang et al. 2004; Struhl 2007; Palazzo and Lee 2015). Indeed, our findings indicate lncRNAs are associated with chromatin marks characteristic of enhancer elements. This close association of lncRNAs with active enhancers may clarify several observations widely construed as suggestive of biological function. These include the widespread sequence conservation of lncRNA promoter regions (The FANTOM Consortium and RIKEN Genome Exploration Research Group and Genome Science Group 2005; Guttman et al. 2009; Derrien et al. 2012; Chen et al. 2016), strong cell type– and developmental stage–specific expression (Cabili et al. 2011; Derrien et al. 2012; Djebali et al. 2012), and phenotypic changes observed following ablation of lncRNA loci (Sauvageau et al. 2013; Dimitrova et al. 2014; Hacisuleyman et al. 2014). Sequence conservation of enhancer regions and their regulation of cell type–specific transcriptional control are well-documented (Heinz et al. 2015; Roadmap Epigenomics Consortium et al. 2015). Conservation of sequence immediately adjacent to lncRNA transcription start sites, previously viewed as lncRNA promoters, may alternatively be interpreted as conserved enhancer regions. Similarly, the characteristic tissue-restricted expression of lncRNAs may reflect activity of the adjacent enhancer. Phenotypes observed following ablation of lncRNA loci may equally be due to loss of underlying regulatory DNA regions, as was recently observed to be the case for a number of zebrafish lncRNAs (Goudarzi et al. 2019). Similarly, two recent investigations employing insertion of transcriptional terminator sequences to separate the role of the genomic locus from its RNA products reached similar conclusions (Engreitz et al. 2016; Paralkar et al. 2016). In both cases, *cis* elements were identified as functional, whereas the associated lncRNAs were dispensable.

However, the observations presented in this study do not preclude the possibility of lncRNA *cis*-regulatory roles that occur following the activation of gene expression. A protein-coding gene and lncRNA with a shared promoter region may result in coexpression of both transcripts. Following this, the lncRNA transcript may interact with the shared promoter, affecting both mRNA and lncRNA expression at the same time. In this scenario, due to the shared promoter region, both transcripts could remain coexpressed with the same dynamics. However, synchronous activation was observed between pairs separated by genomic distances that far exceed the boundaries of both human and mouse gene promoters. This suggests that functional *cis*-regulatory roles that rely on coexpression from shared promoter regions are unlikely to account for the observed lncRNA expression dynamics. However, the potential functional consequences of lncRNA autoregulation are intriguing and warrant examination in future studies. Importantly, our observations also do not preclude potential *trans*-regulatory functions, unrelated to activation of adjacent gene expression.

Further, this study focuses on a narrow range of cell types and biological stimuli. Additional studies of other dynamic processes, such as cellular differentiation, would strengthen the generality of these findings. The current study also relies on bulk RNA-seq measurements which obscure the timing of transcriptional responses within individual cells. Future studies focused on lncRNA dynamics may benefit from inclusion of gene expression measurement at single-cell resolution. Measurement of gene activation dynamics using pre-mRNA also restricts the analysis to genes containing introns.

The findings of this study also provide an additional criterion by which future studies may distinguish subsets of functional noncoding RNAs. If most lncRNAs originate as transcriptional by-products, examples that violate this trend and are transcribed independent of the activity of neighboring protein-coding gene loci may represent functional transcripts. Further research is required to determine whether such independently regulated lncRNAs are associated with characteristics such as localization with chromatin-associated or gene-silencing factors, increased abundance, stability, or sequence-level conservation that may indicate a subset of functional lncRNAs.

## Methods

### Cell culture and RNA extraction

Human glioblastoma T98G cells obtained from the American Type Culture Collection were cultured in Gibco Dulbecco's Modified Eagle Medium (DMEM) supplemented with 10% fetal calf serum (FCS) at 37°C in humidified atmosphere with 5% CO_2_. For each time point, two million cells were seeded and allowed to equilibrate for 24 h, followed by a 72-h incubation in serum-free DMEM. Cells were stimulated with 20% FCS/DMEM at specified time points, lysed with TRIzol reagent (Ambion), homogenized, and frozen for subsequent RNA isolation. RNA extraction and purification was performed using a miRNeasy Mini kit and RNase-free DNase (Qiagen).

### RNA sequencing

RNA samples were depleted of ribosomal RNA (rRNA) using Ribo-Zero biotinylated, target-specific oligos (Illumina) combined with RNAClean XP beads (Beckman Coulter). Following purification, rRNA-depleted samples were prepared for sequencing using an Illumina TruSeq Stranded Total RNA library prep kit. After individual library QC, the sample pool size and concentration were determined using a LabChip GX DNA High Sensitivity assay and qPCR using a KAPA Library Quantification kit (Roche). Uniquely indexed samples were pooled in equimolar concentration, diluted and denatured as one, clustered across eight flow cell lanes, and sequenced at 125-bp paired-end resolution using an Illumina HiSeq 2500 v4.0 sequencing system to provide a mean sequencing depth of 37.2 million reads per time point sample.

### Bioinformatic analysis

In addition to the descriptions provided below, all code used to produce the presented analyses and figures, along with links to external data sets, are provided in the associated GitHub repository (https://github.com/WalterMuskovic/lncRNA_time_course).

### RNA sequencing data analysis

Sequencing data for the mouse dendritic cell LPS response time course were obtained from NCBI Gene Expression Omnibus (GEO; https://www.ncbi.nlm.nih.gov/geo/) under accession GSE56977. A detailed description of the sample preparation and sequencing can be found in the associated publication (Rabani et al. 2014). Both human glioblastoma T98G and mouse time course reads were trimmed to remove Illumina adapter sequences, with cutadapt, version 1.11 (Martin 2011). Trimmed reads were aligned to the GRCh38 and GRCm38 primary genome assemblies using STAR (Dobin et al. 2013), version 2.5.2a. Aligned reads from all time points were combined for de novo transcriptome assembly with StringTie, version 2.1.3 (Pertea et al. 2015). Subsequent statistical analysis was performed with R (R Core Team 2020). Read counts were quantified for each time point using the Rsubread R package (Liao et al. 2014), version 1.34.6. Counts were normalized using the median of ratios method implemented in the DESeq2 R package (Love et al. 2014), version 1.24.0. For each gene, the transcript with the highest (length-adjusted) counts was selected. To identify human and mouse genes activated in response to serum stimulation, each gene was tested for autocorrelation using a Ljung–Box test with the stats R package, version 4.0.2. Genes with an adjusted *P*-value cut-off below 0.01 were retained, following correction for multiple-testing with Benjamini-Hochberg adjustment. To assist visualization, protein-coding genes and lncRNAs with similar expression profiles were grouped by *k*-means cluster analysis. To determine the optimal cluster number (k), the total within-cluster sum of squares (WSS) was calculated for a range of values of k. Examining a curve of WSSs according to the number of clusters k, a value was chosen such that adding additional clusters did not greatly reduce the total intra-cluster variation. For all transcript classes, a value of k = 6 was determined to be appropriate.

### Inference of transcript-specific half-lives

Following the method described by Zeisel et al. (2011), we model transcription dynamics with the following differential equation:

$$\frac{dM}{dt}=\text{β}P(t)-\alpha M(t),$$

in which the rate change in mRNA concentration $\left(\frac{dM}{dt}\right)$ corresponds to the balance between transcription and degradation. *β* denotes the splicing rate coefficient of the pre-mRNA *P*(*t*) to mature mRNA *M*(*t*), which degrades at a rate captured by *α*. Transcript-specific mRNA half-lives are given by $T_{1/2}=\frac{ln2}{\alpha }$. To determine the time-invariant model parameters (*β* and *α*), normalized mRNA and pre-mRNA counts were fit using least squares. Pre-mRNA expression was captured using only reads mapped to the last 10 kb of a gene's introns. This was done to remove the effects of transcription delays due to gene length. We note that, for all other analyses, pre-mRNA expression was measured using the first 10 kb of intron sequence from the TSS, excluding any exon sequences. Model parameters were selected as those minimizing the difference between model predictions of mRNA dynamics relative to measured levels.

### Impulse model fits to time course data

To assist with visualization, lines were fit to the pre-mRNA profiles presented in the upper panels of Figure 2 and the first/last 10 kb of pre-mRNA presented in Figure 3. Fits were obtained using the parametric impulse model described by Chechik and Koller (2009), designed to capture gene expression responses that exhibit an abrupt early response before settling at a second steady-state level. The six-parameter model function described by Chechik and Koller (2009)

$$f(t)=\frac{1}{h_{1}}\left(h_{0}+(h_{1}-h_{0})\times \frac{1}{1+e^{-\lambda (t-t_{1})}}\right)\times \left(h_{2}+(h_{1}-h_{2})\times \frac{1}{1+e^{\lambda (t-t_{2})}}\right)$$

describes two transitions, both with the same slope, captured by *λ*. We generalized the model slightly to allow two transitions with different slopes, defined by *λ*_1_ and *λ*_2_:

$$f(t)=\frac{1}{h_{1}}\left(h_{0}+(h_{1}-h_{0})\times \frac{1}{1+e^{-\lambda_{1}(t-t_{1})}}\right)\times \left(h_{2}+(h_{1}-h_{2})\times \frac{1}{1+e^{\lambda_{2}(t-t_{2})}}\right).$$

Optimal model parameters were determined by least squares, minimizing the sum of squared error between the impulse model fit and measured pre-mRNA levels.

### Roadmap Epigenomics Project and ENCODE chromatin-accessibility and histone modification data

The DNase-seq and histone modification ChIP-seq data for GRCh38 genomic regions presented in Figure 5 were obtained from the NIH Roadmap Epigenomics Project (Roadmap Epigenomics Consortium et al. 2015). Data from genomic regions of interest were extracted from genome-wide −log_10_(*P*-value) signal tracks containing uniformly processed data from 111 consolidated epigenomes, representing a diverse range of human cell types and tissues (Roadmap Epigenomics Consortium et al. 2015). To classify lncRNAs as enhancer-associated or promoter-associated, the Search Candidate *cis*-Regulatory Elements by ENCODE (SCREEN) registry of candidate *cis*-Regulatory Elements (cCREs) v3 was used (The ENCODE Project Consortium et al. 2020). SCREEN cCREs are classified as promoter-like or enhancer-like based on the presence of strong DNase and H3K4me3 signals versus strong DNase and H3K27ac but low H3K4me3 signal, respectively. lncRNAs were classified based on the presence of a cCRE within 300 bp of their transcription start site.

### Block bootstrap

We sought to assess whether coding/lncRNA pairs that are close together are more correlated in their expression profiles than would be expected by chance by plotting a simulation envelope around the relationship between Pearson's correlation and separation distance to show the 1st and 99th percentiles under the null hypothesis. If the trend is outside the simulation envelope, then it indicates there is a relationship between the two that is beyond what is expected by chance. A naive method for the simulation envelope involves creating pseudosamples by randomly permuting the separation distances (but not the Pearson correlations) and using these to recreate the “null” trend—where coding/lncRNA correlation and separation distance are not correlated. However, both classes of transcripts are spatially correlated (Supplemental Fig. 3) and naive permutation would ignore this dependence. Hence, a block bootstrap approach was employed to create the pseudosamples for the simulation envelope (Lahiri 2013). To perform the block bootstrap, pseudochromosomes were created by splitting chromosomes into sublengths of a determined block size for each transcript class. Sublengths were then sampled with replacement to obtain the pseudochromosomes, with a GAM subsequently fit to the trend in Pearson correlation versus separation distance on all the coding/lncRNA pairs in the pseudochromosome. A simulation envelope was obtained by taking the 1st and 99th percentiles from 1000 iterations of the block bootstrap. A schematic of the method along with the code used to implement it is provided in the accompanying GitHub repository. To determine the appropriate block size for each transcript class, separation distances were randomly shuffled 1000 times and generalized additive models were fit to the relationship between distance and correlation to obtain 1st and 99th quantiles. The distance at which the GAM fit to the unpermuted data exceeded the 99th quantile was taken as the block size, so that the expression profiles between sublengths of chromosome could be considered approximately independent.

### Cross-correlation

The ccf function from the R stats package, version 3.6.1, was used to compute the cross-correlation between lncRNA and coding expression profiles, with time lags ranging from −200 to 200 min for the T98G time course and from −90 to 90 min for the mouse LPS time course. The lncRNA expression profile is lagged, while the coding gene expression profile is held constant. To negate any effects of transcription delays due to gene length or transcript half-lives, coding gene pre-mRNA and lncRNA expression was calculated using only the first 10 kb of intron regions. The mean was taken for all coding/lncRNA pairs within the specified separation distance. To gain an estimate of uncertainty in the trend (accounting for autocorrelation in expression profiles along the chromosome), the above procedure was repeated 1000 times on pseudochromosomes generated using the block bootstrap method, from which the 1st–99th quantiles were obtained in each separation distance category.

## Data access

All raw and processed sequencing data generated in this study have been submitted to the NCBI Gene Expression Omnibus (GEO; https://www.ncbi.nlm.nih.gov/geo/) under accession number GSE138662. All code used to produce the analysis presented in this work are available in the GitHub repository (https://github.com/WalterMuskovic/lncRNA_time_course) and as Supplemental Code.

## Supplementary Material

Supplemental Material
