# Supplementary material for: High temporal resolution RNA-seq time course data reveals widespread synchronous activation between mammalian lncRNAs and neighboring protein-coding genes
Source: Genome Res. 2022 Aug;32(8):1463–73. doi: 10.1101/gr.276818.122 (PMC9435739; doi:10.1101/gr.276818.122)
Supplement: Supplemental Material [file supp_32_8_1463__DC1.html]

High temporal resolution RNA-seq time course data reveals widespread synchronous activation between mammalian lncRNAs and neighboring protein-coding genes — High temporal resolution RNA-seq time course data reveals widespread synchronous activation between mammalian lncRNAs and neighboring protein-coding genes — Supplemental Material 

# High temporal resolution RNA-seq time course data reveals widespread synchronous activation between mammalian lncRNAs and neighboring protein-coding genes

## Supplemental Material

- Supplemental\_Fig\_S1.pdf
- Supplemental\_Fig\_S2.pdf
- Supplemental\_Fig\_S3.pdf
- Supplemental\_Fig\_S4.pdf
- Supplemental\_Fig\_S5.pdf
- Supplemental\_Code.zip
