# Supplementary material for: High temporal resolution RNA-seq time course data reveals widespread synchronous activation between mammalian lncRNAs and neighboring protein-coding genes
Source: Genome Res. 2022 Aug;32(8):1463–73. doi: 10.1101/gr.276818.122 (PMC9435739; doi:10.1101/gr.276818.122)
Supplement: Supplemental Material [file supp_gr.276818.122_Supplemental_Fig_S1.pdf]

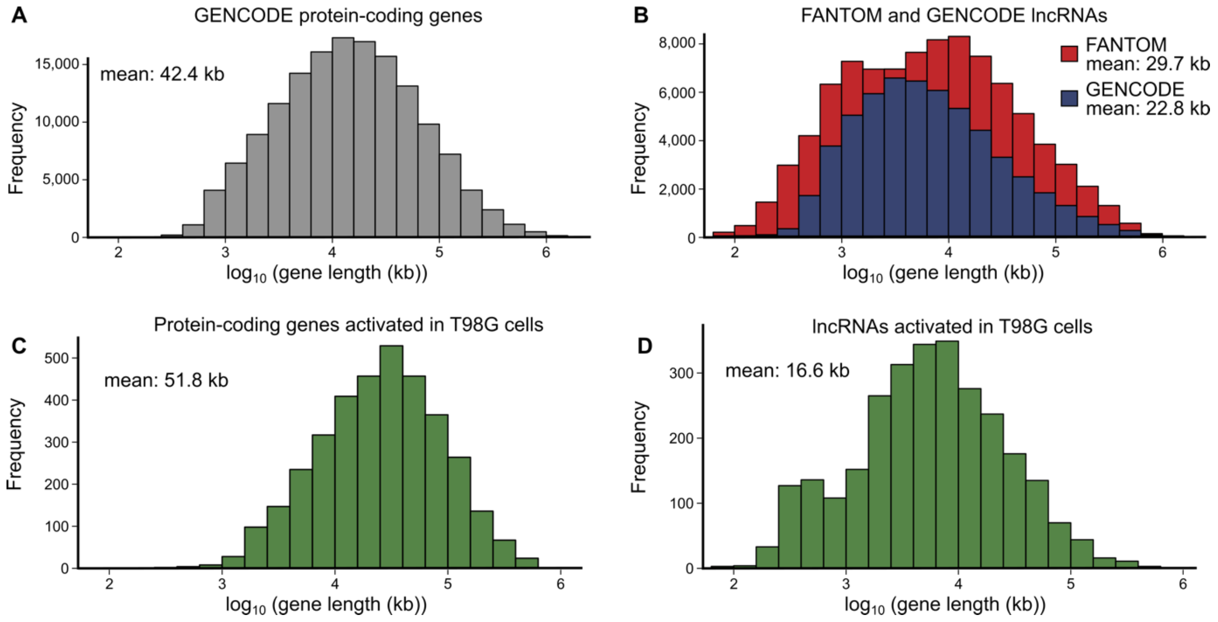

Supplementary Figure 1. lncRNA and protein-coding gene length

A, Histogram showing the distribution of lengths for all protein-coding transcripts in the GENCODE Human Release 29 annotation. **B**, Lengths of all lncRNA transcripts in the FANTOM CAGE associated transcriptome and GENCODE Human Release 29 annotations. **C**, Lengths of all protein-coding genes and **D**, lncRNAs activated in human T98G cells in response to serum stimulation.
