# Supplementary material for: High temporal resolution RNA-seq time course data reveals widespread synchronous activation between mammalian lncRNAs and neighboring protein-coding genes
Source: Genome Res. 2022 Aug;32(8):1463–73. doi: 10.1101/gr.276818.122 (PMC9435739; doi:10.1101/gr.276818.122)
Supplement: Supplemental Material [file supp_gr.276818.122_Supplemental_Fig_S2.pdf]

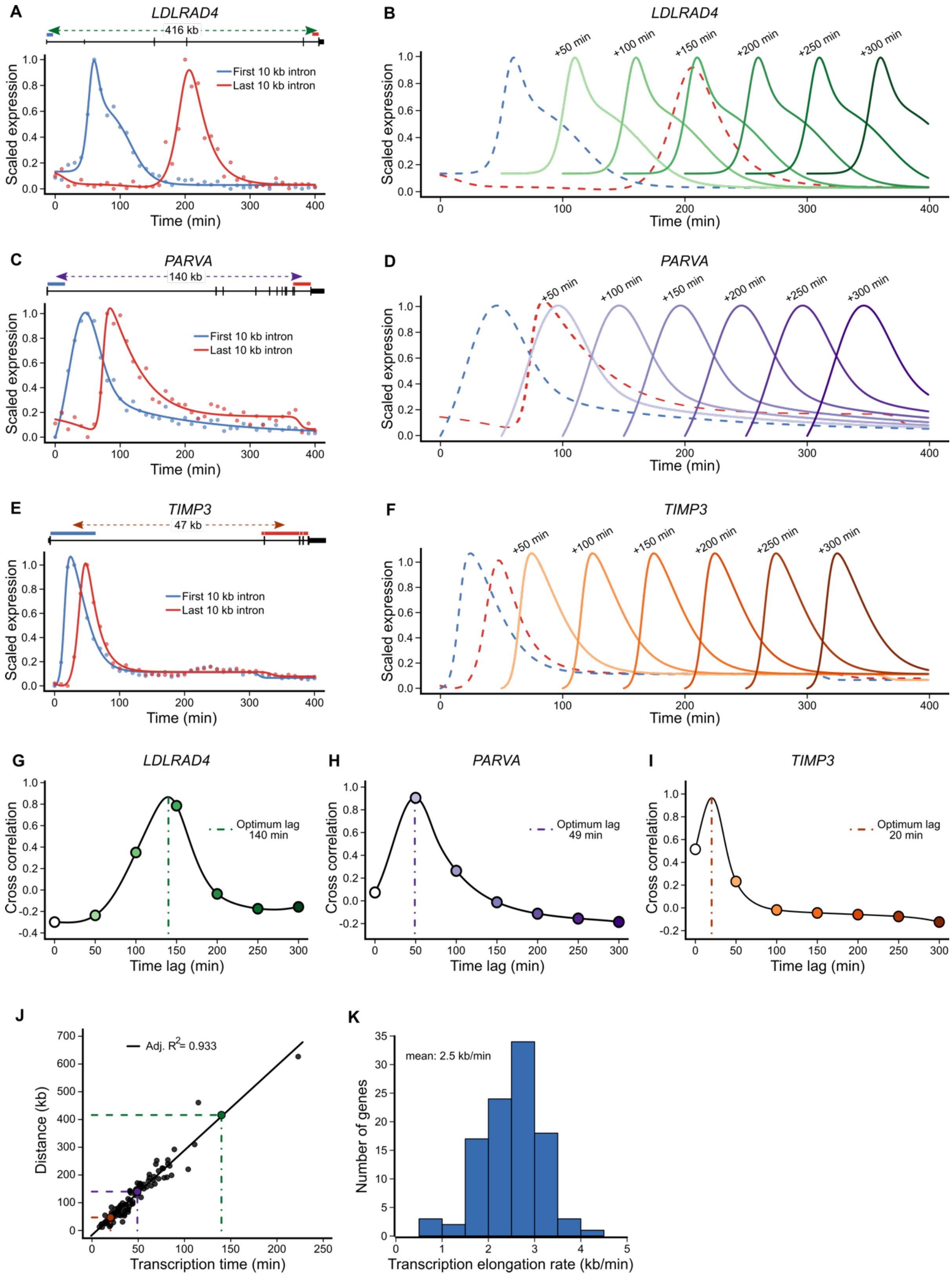

Supplementary Figure 2. Estimation of the human RNA polymerase II transcription elongation rate

A,**C**,**E**, Expression dynamics of the first and last 10 kb of pre-mRNA for three genes of different length. Lines represent impulse model fits to the normalized expression estimates obtained through RNA-seq (points). Included above are schematic illustrations of the three genes *LDLRAD4*, *PARVA* and *TIMP3*, blue and red horizontal bars indicating the regions of intron used to quantify the first and

last 10 kb of pre-mRNA respectively. Distance labels indicate the distance between the centers of the first/last 10 kb intervals. **B**,**D**,**F**, Impulse model fits to the pre-mRNA expression dynamics of the three genes as in **A**, **C** and **E** with time-lagged copies of the first 10 kb of pre-mRNA overlaid at intervals of 50 min. **G**-**I**, Lagged correlations between the first and last 10 kb of each gene's pre-mRNA, obtained by keeping the expression profile of the last 10 kb of pre-mRNA constant and shifting the expression profile of the first 10 kb of pre-mRNA from 0 to 300 min. Filled circles correspond to the time lags overlaid in **B**, **D** and **F**. Vertical lines indicate the time lag at which the correlation between the expression profile of the last 10 kb of pre-mRNA and the lagged expression profile of the first 10 kb of pre-mRNA is maximal. **J**, Scatterplot of the relationship between transcription time and genomic distance with linear model fit overlaid. Colored circles correspond to the transcription times and distances of the three genes presented in **A**-**I**. **K**, Histogram showing the distribution of transcription elongation rates.
