# Supplementary material for: High temporal resolution RNA-seq time course data reveals widespread synchronous activation between mammalian lncRNAs and neighboring protein-coding genes
Source: Genome Res. 2022 Aug;32(8):1463–73. doi: 10.1101/gr.276818.122 (PMC9435739; doi:10.1101/gr.276818.122)
Supplement: Supplemental Material [file supp_gr.276818.122_Supplemental_Fig_S3.pdf]

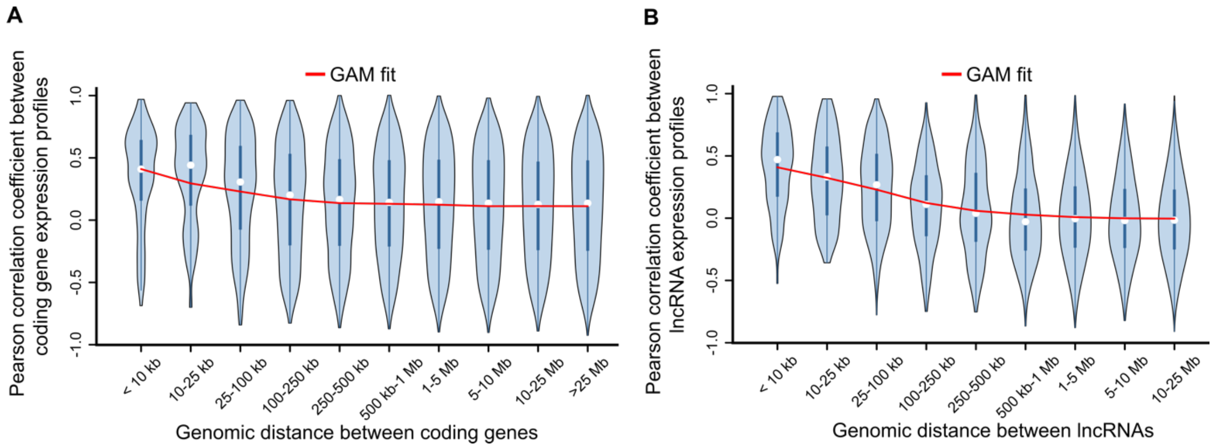

Supplementary Figure 3. Correlated expression amongst adjacent protein-coding genes and lncRNAs

A, Violin plot of Pearson correlation coefficients between protein-coding gene expression profiles, binned by the genomic distance between genes. The overlaid generalized additive model (GAM) fit summarizes the trend between distance and pre-mRNA expression correlation between coding gene pairs (e.d.f=7.703, P<2e-16). **B**, Violin plot of Pearson correlation coefficients between lncRNA expression profiles, binned by the genomic distance between lncRNAs. The overlaid generalized additive model (GAM) fit summarizes the trend between distance and lncRNA expression correlation between lncRNA pairs (e.d.f=8.969, P<2e-16).
