# Supplementary material for: High temporal resolution RNA-seq time course data reveals widespread synchronous activation between mammalian lncRNAs and neighboring protein-coding genes
Source: Genome Res. 2022 Aug;32(8):1463–73. doi: 10.1101/gr.276818.122 (PMC9435739; doi:10.1101/gr.276818.122)
Supplement: Supplemental Material [file supp_gr.276818.122_Supplemental_Fig_S4.pdf]

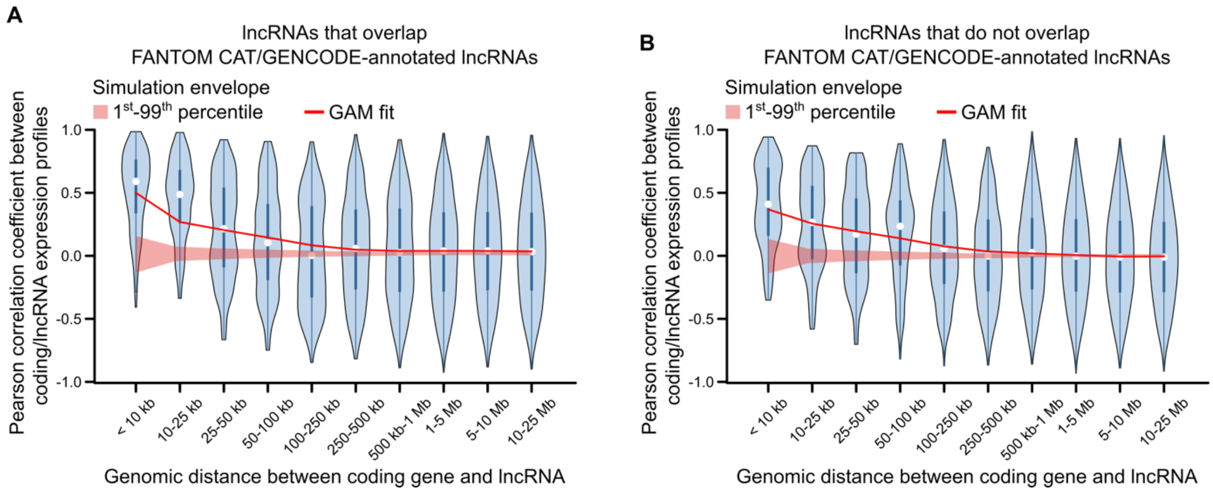

Supplementary Figure 4. Correlated expression amongst protein-coding genes and lncRNAs with and without overlap of annotated lncRNAs

A, Violin plot of Pearson correlation coefficients between the expression profiles of protein-coding genes and lncRNAs that overlap GENCODE/FANTOM CAT-annotated lncRNAs, binned by genomic distance. The overlaid GAM fit summarizes the trend between distance and expression correlation between coding gene/lncRNA pairs (e.d.f=7.851, P<2e-16). **B**, Violin plot of Pearson correlation coefficients between expression profiles of protein-coding gene and lncRNAs that do not overlap annotated lncRNAs. As in **A,** the GAM fit summarizes the trend between distance and expression correlation of the coding gene/lncRNA pairs (e.d.f=7.964, P<2e-16). In both **A** and **B**, a simulation envelope, generated using a block-bootstrap approach (see Methods), demonstrates the expected trend under the null hypothesis that distance and correlation are unrelated. For both novel and annotated lncRNAs, the trend in correlation against separation distance lies well outside the simulation envelope indicating a relationship unlikely to be due to chance.
