# Supplementary material for: High temporal resolution RNA-seq time course data reveals widespread synchronous activation between mammalian lncRNAs and neighboring protein-coding genes
Source: Genome Res. 2022 Aug;32(8):1463–73. doi: 10.1101/gr.276818.122 (PMC9435739; doi:10.1101/gr.276818.122)
Supplement: Supplemental Material [file supp_gr.276818.122_Supplemental_Fig_S5.pdf]

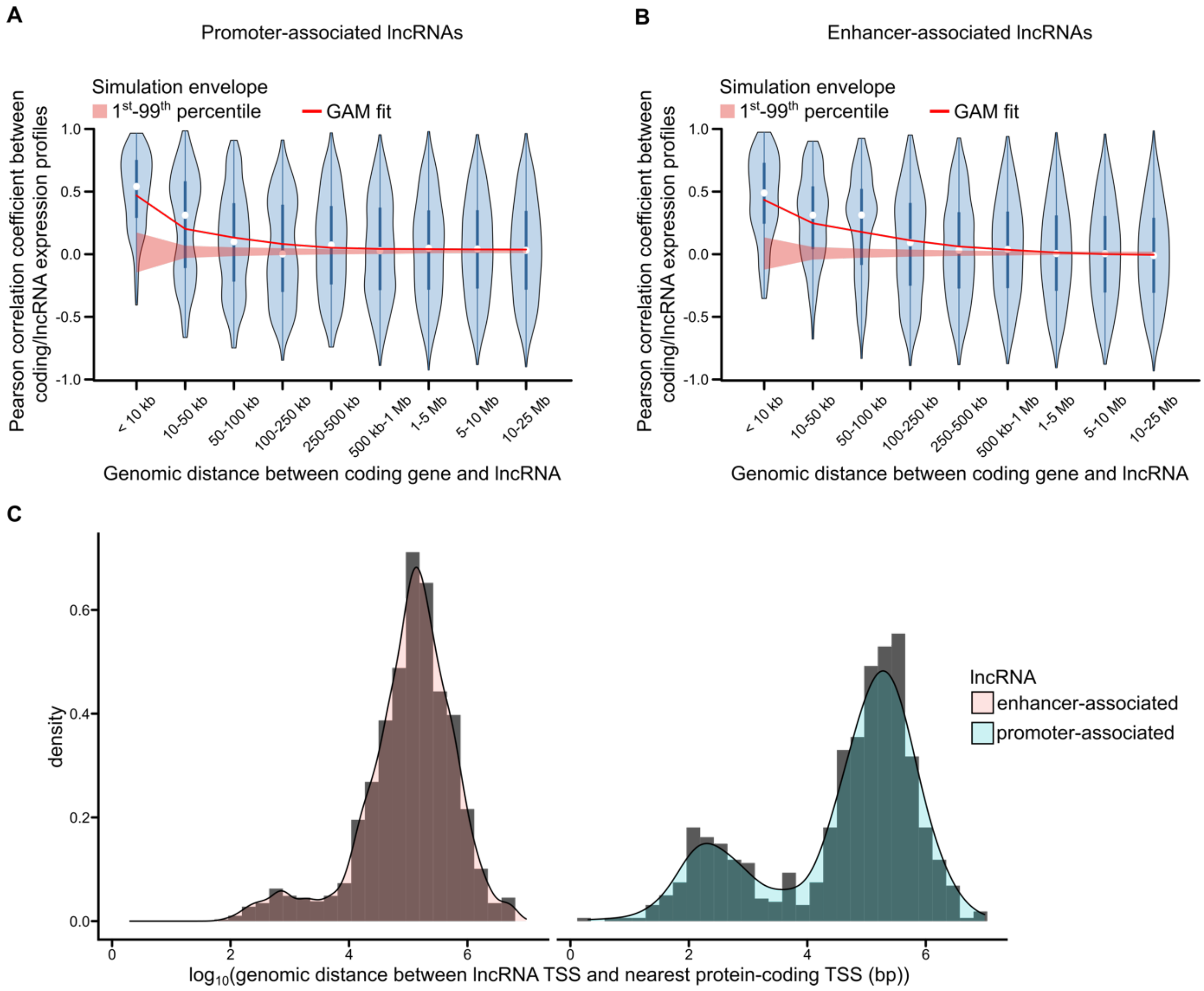

Supplementary Figure 5. Correlated expression amongst protein-coding genes and lncRNAs associated with promoter or enhancer *cis*-regulatory elements

A, Violin plot of Pearson correlation coefficients between the expression profiles of protein-coding genes and lncRNAs associated with *cis*-regulatory elements with promoter-like signatures, binned by genomic distance. The overlaid GAM fit summarizes the trend between distance and expression correlation between coding gene and promoter-associated lncRNA pairs (e.d.f=6.492, P<2e-16). **B**, Violin plot of Pearson correlation coefficients between expression profiles of protein-coding gene and enhancer-associated lncRNAs. As in **A,** the GAM fit summarizes the trend between distance and expression correlation of the coding gene and enhancer-associated lncRNA pairs (e.d.f=6.733, P<2e-16). In both **A** and **B**, a simulation envelope, generated using a block-bootstrap approach (see Methods), demonstrates the expected trend under the null hypothesis that distance and correlation are unrelated. For both promoter-associated and enhancer-associated lncRNAs, the trend in correlation against separation distance lies well outside the simulation envelope indicating a relationship unlikely to be due to chance. **C,** Distance between the TSS of each enhancer-associated and promoter-associated lncRNA and the TSS of the nearest activated protein-coding gene.
